# Simvastatin Restores HDAC1/2 Activity and Improves Behavioral Deficits in Angelman Syndrome Model Mouse

**DOI:** 10.3389/fnmol.2019.00289

**Published:** 2019-11-26

**Authors:** Vipendra Kumar, Tripti Joshi, Naman Vatsa, Brijesh Kumar Singh, Nihar Ranjan Jana

**Affiliations:** ^1^Cellular and Molecular Neuroscience Laboratory, National Brain Research Centre, Gurgaon, India; ^2^School of Bioscience, Indian Institute of Technology, Kharagpur, India

**Keywords:** Angelman syndrome, UBE3A, simvastatin, HDAC1/2, BDNF

## Abstract

Angelman syndrome (AS) is a neurodevelopmental disorder categorized by severe disability in intellectual functions and affected by the loss of function of maternally inherited *UBE3A* gene. Mice deficient for the maternal *Ube3a* recapitulates many distinguishing behavioral features of the AS and is used as a typical model system to understand the disease pathogenic mechanism. Here, we first show a significant increase in HDAC1 and HDAC2 activities in AS mice brain from as early as embryonic day 16(E16). In depth study further reveals that the deficiency of Ube3a leads to transcriptional up-regulation of both HDAC1 and HDAC2. Restoration of HDAC1 and HDAC2 activities (as evident from the increased acetylation of histones H3 and H4) using simvastatin significantly improves the cognitive deficit and social interaction behavior in AS mice. Simvastatin treatment also restores the reduced level of BDNF in AS mice brain. Finally, we demonstrate that the treatment of simvastatin to primary cortical neuronal culture prepared from AS mice embryo also rescues altered acetylation of histones H3 and H4 and the level of BDNF. These results suggest that simvastatin could be a promising drug for the treatment of AS.

## Introduction

Angelman syndrome (AS) is a neurodevelopmental disorder usually characterized by severe delay in developmental landmarks, intellectual disability, ataxic gait, impaired speech and epilepsy. AS individuals also recurrently exhibit uncontrolled laughter (for which it was initially named as happy puppet syndrome) along with flappy hand movement and dys-regulated sleep patterns (Williams et al., 2006, 2010). Genomic studies find that AS is predominantly caused due to the large deletion of 15q11-q13 region in the maternal chromosome. Loss of function mutations in the *UBE3A* gene (situated within 15q11-q13 locus) also reported in a subcategory of AS patients (Albrecht et al., 1997; Kishino et al., 1997; Matsuura et al., 1997; Fang et al., 1999). These findings strongly indicate that *UBE3A* is one of the potential candidate genes for the AS. Furthermore, *Ube3a*-maternal deficient mice (AS mice) reproduce many behavioral deficits that are observed in AS patients (Jiang et al., 1998; Heck et al., 2008; Mulherkar and Jana, 2010; Shi et al., 2015). Interestingly, *UBE3A* gene is paternally imprinted in the neuron (Albrecht et al., 1997; Yamasaki et al., 2003). Therefore, loss of function mutations in the maternal *UBE3A* gene could lead to its complete absence of expression in neurons.

*UBE3A* gene encodes for a 100 kDa globular protein known as E6AP/UBE3A, which is initially characterized as a E3 ubiquitin ligase that selectively targets wide range of cellular proteins for their ubiquitination and subsequent proteasomal degradation (Huibregtse et al., 1995). UBE3A also acts as a co-activator of steroid hormone receptors and regulates the expression of their target genes (Ramamoorthy and Nawaz, 2008). Increasing evidence now indicates that the ubiquitin ligase function of Ube3a is crucial in regulating synapse development and synaptic function (Greer et al., 2010; Pignatelli et al., 2014; Sun et al., 2015, 2018; Kim et al., 2016). AS mice also exhibit impaired activity-driven dendritic spine maintenance in hippocampal CA1 as well as cortical layer III and V pyramidal neurons (Kim et al., 2016). Further studies reveal that the absence of Ube3a leads to aberrant increase in the level of activity-regulated cytoskeletal associated protein (Arc), Ephexin5 (a RhoA guanine nucleotide exchange factor) and a small conductance calcium-activated potassium channel (SK2), which might be linked with altered excitatory synaptic transmission, synapse formation and experience-dependent synaptic remodeling observed in AS mice (Yashiro et al., 2009; Greer et al., 2010; Margolis et al., 2010; Sato and Stryker, 2010; Sun et al., 2015).

Although, substantial progress have been made in understanding the pathogenic mechanism of AS, presently there is no actual therapy. The reactivation of dormant paternal allele of *UBE3A* is being considered one of the promising therapeutic strategies (Malpass, 2012). In one study, topoisomerase inhibitors are revealed to unsilence the paternal *Ube3a* expression by inhibiting the large non-coding antisense RNA transcript (UBE3A-ATS) (Huang et al., 2012). Nonetheless, therapeutic opportunities of these topoisomerase inhibitors in animal models are yet to be understood. In another study, antisense oligonucleotide of UBE3A-ATS is shown to activate the paternal *Ube3a* and subsequently improves the behavioral deficit in AS mice (Meng et al., 2015). Few reports in mice models also indicate that Ube3a replacement at early developmental stage might be crucial in restoring majority of AS phenotypes (Silva-Santos et al., 2015; Gu et al., 2019).

Chromatin remodeling through post-translational modification in histones play a crucial role in modulating synaptic function and plasticity (Graff et al., 2011; Penney and Tsai, 2014; Whittle and Singewald, 2014). Histones acetylation is implicated in increased synapse formation, induction in hippocampal long-term potentiation and memory consolidation (Bousiges et al., 2010; Peleg et al., 2010; Mews et al., 2017). In other studies, histone deacetylase 2 (HDAC2) is reported to negatively regulate the synaptic function and plasticity and consequently influence the memory formation (Guan et al., 2009; Graff et al., 2012). Recently, we observed aberrantly increased HDAC1 and HDAC2 activities in adult AS mice brain, which might be linked with the altered synaptic function and plasticity in these mice (Jamal et al., 2017). However, the mechanistic basis and consequence of increased HDAC1/2 activities are not known. In the present study, we first report that the aberrantly increased HDAC1/2 activities in AS mice brain is observed from early developmental days (as early as from embryonic days 16). Subsequently, we find that Ube3a is not involved in the degradation of HDAC1/2 rather it regulates their transcription. Up-regulation of HDAC1/2 activities in AS mice brain prompted us to investigate the effect of HDAC1/2 inhibitor in rescuing of behavioral deficits in these mice. We have chosen simvastatin, because this FDA approved brain penetrating drug not only inhibits HDAC1/2 activities but also induces the expression of neurotropic factor BDNF apart from its popularly known HMG CoA reductase enzyme inhibition (Lin et al., 2008; Roy et al., 2015). We find that oral administration of simvastatin partially restored altered HDAC1/2 activities and BDNF level in AS mice along with significantly improvement of their behavioral deficits.

## Materials and Methods

### Materials

Cell culture reagents including high glucose DMEM (Dulbecco’s Modified Eagle Medium), (β-D-arabinofuranosyl cytosine, D-glucose, ploy L-lysine, BCA protein estimation kit), and primary antibodies such as mouse monoclonal anti-β-actin (A5316), anti-Ube3a (E8655), and anti-Tuj1 (T8660) were purchased from Sigma. Fetal bovine serum (FBS), trypsin (0.25%), penicillin-streptomycin, neurobasal medium, minimum essential medium, Lipofectamine^®^2000, Opti-MEM, sodium pyruvate were procured from Gibco/Thermo Fisher Scientific. Mouse monoclonal anti-Ube3a was purchased from Santa Cruz Biotechnology (SC-16689) and BD Bioscience (611416). Rabbit monoclonal anti-H3(4499), anti-H3(K9) (9649), anti-H4(13919), anti-H4(K12) (2591) and anti-RAD23A; mouse monoclonal anti-HDAC1(5356) and anti-HDAC2(5113) were purchased from Cell Signaling Technology. Rabbit polyclonal anti-BDNF (NBP1-59304) was from Novus Biologicals and anti-ubiquitin (Z0458) was from DAKO. Horseradish peroxidase (HRP) conjugated and biotinylated secondary antibodies, VectaStain ABC kit, ImmPACT NovaRED HRP substrate kits were purchased from Vector laboratories. Ube3a siRNA and control siRNA were procured from Santa Cruz Biotechnology.

### Animals and Treatment

Ube3a-maternal deficient heterozygous mice (*Ube3a*^*m–/p+*^, AS mice) were purchased from Jackson laboratory (Jackson code:129-Ube3atm1Alb/J) and maintained in standard cages with adequate supply of pelleted food and water. Animals used in all experiments were approved by the Institutional Animal and Ethics Committee (IAEC) of National Brain Research Centre. Committee for the Purpose of Control and Supervision of Experiments on Animals (CPCSEA) guidelines were strictly followed while handling and sacrificing of experimental animals. Ube3a-maternal deficient females (*Ube3a*^*m–/p+*^) were bred with wild-type males to obtain *Ube3a*^*m–/p+*^ mice along with wild type (*Ube3a*^*m+/p+*^). Genotyping of the pup was done as previously described (Jiang et al., 1998) after isolation of genomic DNA from the tail using genomic DNA isolation kit (MDI, India). For simvastatin treatment, adult (P120) male wild type and AS mice were used. Animals were administered either simvastatin (1 mg/kg body weight daily in 3% DMSO) or vehicle (3% DMSO) orally using oral gavage for 60 days. Behavioral tests were performed between 45 and 55 days of treatment.

### Cell Culture, Transfection and Chase Experiments

HT22 cells (Kindly provided by Dr. Dave Schubert, Salk Institute, United States) were cultured in DMEM supplemented with 10% heat inactivated FBS along with penicillin-streptomycin. For simvastatin treatment, cells were cultured in 6-well tissue cultured plate at sub confluent density and after 24 h of platting, cells were treated with various doses of simvastatin for 12 h. In some experiment, cells were transiently transfected with control and Ube3a siRNA using Lipofectamine^®^2000 as per manufacturer’s protocol. After 24 h of transfection, cells were treated with vehicle (DMSO) or simvastatin for 12 h. At the end of the experiment, cells were collected and processed for either immunoblot analysis or subjected to RNA extraction followed by RT-qPCR. In cycloheximide chase experiment, cells were transfected with control and Ube3a siRNA as above. Twenty four hours of post transfection, cells were chased with cycloheximide (25 μg/ml) for different time periods. Collected cells were then subjected to immunoblot analysis using various antibodies.

### HDAC Activity Assay

Mouse cortical samples or HT22 cells were lysed in lysis buffer (10 mM Tris-pH7.5, 10 mM NaCl, 15 mM MgCl_2_, 250 mM Sucrose, 0.5% NP-40, 0.1 mM EGTA and complete protease inhibitor cocktail) and briefly sonicated on ice. Lysates were subsequently passed through sucrose cushion (30% sucrose, 10 mM Tris-pH-7.5, 10 mM NaCl, 3 mM MgCl_2_) at 1,300 × *g* for 10 min in a refrigerated centrifuge. Nuclear pellets were then re-suspended in extraction buffer (50 mM, HEPES-pH 7.5, 420 mM NaCl, 0.5 mM EDTA, 0.1 mM EGTA, 10% glycerol), sonicated and centrifuged at 20,000 × *g* for 10 min. Supernatant containing crude nuclear extract was used for HDAC activity assay using fluorometric HDAC activity assay kit (Abcam, ab156064) according to the manufacturer’s protocol.

### Primary Neuronal Culture

Cortical primary neurons were cultured from E16 (Embryonic day 16) embryos of pregnant AS dams. Pregnant dams were sacrificed, cortices were dissected out from embryos and trypsinized at 37°C in the dissection medium containing Hank’s balanced salt solution (97.5%), glucose (0.1%), sodium pyruvate (0.11 mg/ml), 10 mM HEPES (pH 7.3), trypsin (0.25%) and DNase 1 (1.2 unit/ml). Detached cells were plated (300 cells/mm^2^) onto poly L-lysine (1 mg/ml) coated coverslips. The plating medium contains minimum essential medium (86%), heat inactivated FBS (10%), glucose (0.45%) as well as sodium pyruvate, glutamine and antibiotics. After 15 h of plating, medium was replaced by neuronal maintenance medium containing neurobasal medium (96%), B-27 supplement including glutamine and antibiotics. Beta-D-arabinofuranosyl cytosine (3 μM) was added to the cultured media for 24 h to arrest glial proliferation. At every 3 days, half of the media was replaced with fresh neuronal maintenance medium. At DIV14 (days *in vitro* 14), neurons were treated with either simvastatin or vehicle (DMSO) and subsequently processed for immunoblotting or immunofluorescence staining experiments.

### Immunoblotting Experiments

Mice were sacrificed, brain samples (cortex and hippocampus) were dissected out and snap frozen in liquid nitrogen followed by storage at −80°C. Tissues were lysed in RIPA lysis buffer (10 mM Tris, pH 7.4, 150 mM NaCl, 2.5 mM EGTA, 10 mM EDTA, 0.1% SDS, 1% Triton X-100, 1% sodium deoxycholate, 0.1 mM Na_2_VO_5_, 10 mM NaF, 5 mM Na_4_P_2_O_7_, and complete protease inhibitor cocktail) at 4°C using tissue homogenizer. Lysates were sonicated and centrifuged at 15,000 × *g* for 10 min. Supernatants were collected and protein amounts were determined using BCA method and then subjected to SDS-PAGE. Resolved proteins were transferred onto nitrocellulose membrane followed by blocking with 5% non-fat skimmed milk and then subjected to immunoblot analysis. The dilutions of primary antibodies used were as follows: Ube3a, β-actin, total and acetylated histones H3/H4 at 1:5000; RAD23A, ubiquitin, HDAC1 and HDCA2 at 1:2000.

### Immunofluorescence Staining and Confocal Microscopy

Mouse cortical primary neurons derived from wild type and AS mice embryos were cultured up to DIV14. Neurons were treated with either vehicle or 5 μM simvastatin for 12 h. Cells were then washed with PBS, fixed with 4% paraformaldehyde (PFA) for 10 min, washed with PBS, permeabilized with 0.3% triton X-100 for 5 min and blocked for 2 h at room temperature using blocking solution (3% bovine serum, 2% normal goat serum and 0.3% triton X-100 made in PBS). Cells were incubated for 12–16 h at 4°C with primary antibodies (mouse anti-Ube3a 1:500 and rabbit anti-BDNF 1:200 dilutions) diluted in blocking solution followed by washing with PBS and probed with fluorescent-labeled secondary antibodies at room temperature for an hour. Coverslips were mounted using DAPI mounting medium after three washes of PBS to remove excess secondary antibody. Image acquisition was performed using LSM 510 meta confocal microscope.

### Immunohistochemistry

Mice were anesthetized using ketamine (100 mg/kg body weight) and Xylazine (10 mg/kg body weight) and were transcardially perfused using PBS followed by 4% PFA. After perfusion, brains were kept in 4% PFA for 24 h and then subjected for 10, 20, and 30% sucrose gradient for 24 h each at 4°C. Brains were cryo-sectioned using Leica CM3050 cryotome and 20 μm sections were obtained. Sections were stored in PBS containing 0.02% sodium azide at 4°C. For immunohistochemical staining, brain sections were incubated in antigen unmasking solution at 70°C for 40 min for antigen retrieval and after that sections were washed with PBS and endogenous peroxidases were quenched using quenching solution (10% H_2_O_2_, 10% methanol in PBS) for 15 min followed by washing with PBS. Sections were next permeabilized by 0.3% triton X-100 for 10 min, blocked with 3% normal goat serum, 1% bovine serum albumin and 0.3% triton X-100 for 2 h at room temperature. Sections were probed with the primary antibody diluted in blocking solution and incubated at 4°C overnight. Ube3a antibody (BD bioscience) was used at 1:50 dilutions and histone and their acetylated derivatives specific antibodies were used at 1:500 dilutions. Sections were washed with PBS and incubated with biotinylated secondary antibodies at a dilution of 1:500 for 1 h, washed and incubated with VECTASTAIN-Elite ABC solution for 2 h as per the manufacturer’s protocol. Staining was developed using ImmPACT Novared peroxidase substrate kit. Images were acquired using Leica DM RXA2 bright field microscope.

### RT-qPCR

Total RNA was extracted from cortices of wild type and AS mice brain and HT22 cells using Trizol reagent as per manufacturer’s protocol. The cDNA was synthesized using cDNA synthesis kit (TAKARA, Japan) followed by qPCR for Ube3a, HDAC1 and HDAC2 using Power CYBR green master mix from Applied Biosystems. The reaction was carried out in Applied Biosystems ViiA7 real time PCR machine. Results were normalized with 18S rRNA and expressed in terms of fold change. Primer sequence for HDAC1, HDAC2, Ube3a and 18S were as follows: HDAC1F, 5′-CCATCCTGGAACTGCTAAAG-3′; HDAC1R, 5′-ACCCGGTCTGTAGTATAGAAG-3′; HDAC2F, 5′-GCTTGCCATCCTCGAATTA-3′; HDAC2R, 5′-CATCACGC GA TTGTTGT-3′; Ube3aF, 5′-CATACCTGAGTCCAGCGAA TTA-3′; Ube3aR, 5′-ACGCCAAGTTCGGTTTCT-3′; 18SF, 5′-GAGGGAGCCTGAGAAACGG-3′; 18SR, 5′-GTCGGGAGT GGGTAATTTGC-3′.

### Behavioral Studies

#### Novel Object Recognition Test

Novel object recognition test was carried out in a specified open field box (45 × 45 × 20 cm). The test involves first habituation of mice to explore in empty open field box followed by training session in the same open field box with novel objects. Habituation was carried out for 3 sessions (5 min each) per day for 3 successive days. Mice were then allowed to explore two identical novel objects that were placed into the open field box 12 cm away from each other. Each mouse was given 5 min to explore both objects and then returned back to their home cage. Time spent on each object was recorded by a wall mounted digital video camera. Two hours later, mouse was again placed back to the same open field box having two familiar objects and allowed to explore for 5 min. Subsequently, one of the similar objects was replaced by a novel object and mice were permitted to explore them for 5 min. Time spent on familiar and novel object was recorded. After each session the box was cleaned thoroughly with 70% ethanol to avoid any olfactory cue bias. Preference ratio for the novel object was calculated as time spent on the novel object over total time spent exploring both familiar and novel objects.

#### Social Interaction Test

To investigate the social interaction, Crawley’s sociability and social novelty experiment procedure was used that uses three rectangular chambers (20 × 45 cm) divided by removable transparent wall to allow free access of mice to all three chambers (Kaidanovich-Beilin et al., 2011). Two similar looking cup-like containers were kept in the left and right side of the test chambers to keep naive mouse. These cups were made up of wires in such a way that it can easily house only one mouse, which can easily move around. The test comprised of habituation and two test sessions for 10 min each. During habituation, test mouse was introduced into the central chamber and allowed to explore the chamber freely for 10 min. In the first test session, one wild type mouse (stranger 1) was introduced inside the cup located at the left side chamber, walls between the chambers were removed and the test mouse was allowed to freely explore all three chambers. The interaction time of the test mouse with the empty cup and the stranger 1 was checked for 10 min and recorded using a wall mounted digital video camera. In the second test session, another wild type mouse (stranger 2) was introduced in the cup located at the right side chamber and the test mouse was allow to explore the familiar (stranger 1) as well as unfamiliar (stranger 2) mouse for 10 min. The time spent by test mouse to explore stranger 1 and stranger 2 was recorded. After each test, the chamber was properly cleaned with 70% ethanol to exclude possibility of olfactory cue bias. The time spent by test mouse to explore the stranger 1 over empty cup-like container was expressed as sociability. Whereas, Social novelty was calculated as the time spent on the stranger 2 over stranger 1.

#### Rotarod Test

Rotarod test was carried out to assess motor coordination. Test mouse was allowed to walk on a rotating rod. Mice were trained for 3 consecutive days at 20 rpm speed. Following the training, test was conducted for 3 days with each day having three sessions. Time of stay on the rotating rod was recorded with maximum limit set to 3 min. Average of all three sessions for 3 days were calculated and expressed as latency to fall.

### Statistical Analysis

Statistical analysis was performed using Sigma Stat software. Values were expressed as mean ± SD. Inter-group comparison was done through one way or two way analysis of variance (ANOVA) followed by Holm–Sidac *post hoc* test. In some experiments Student’s *t*-test was used to compare inter groups. *P* < 0.05 was considered statistically significant.

## Results

### AS Mice Exhibit Increased Levels of HDAC1/2 Along With Reduced Acetylation of Histones H3/H4 From Early Developmental Periods

**FIGURE 1:**
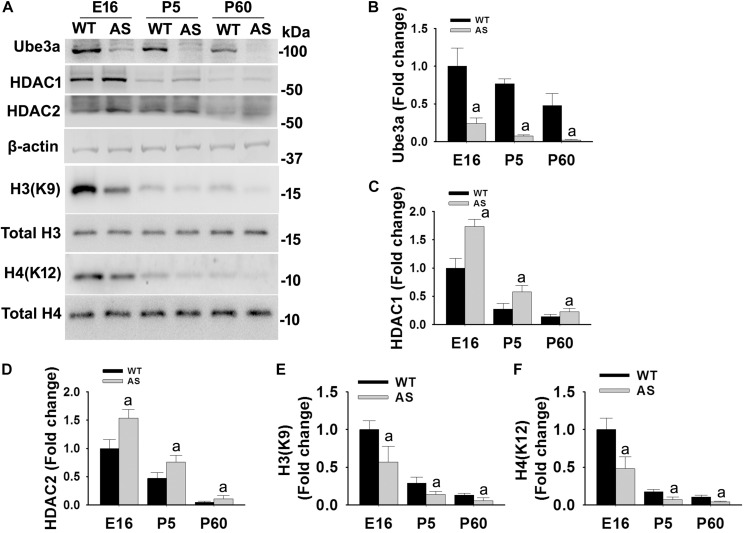
AS mice exhibit increased levels of HDAC1 and HDAC2 along with hypo-acetylation of histones H3(K9) and H4(K12) from early developmental days. **(A)** Cortical brain regions obtained from both wild type and AS mice at their different developmental stages (E16, P5, and P60) were processed for immunoblot analysis using antibodies against HDAC1 and HDAC2, various histones and their acetylated derivatives. **(B–F)** Densitometric analysis of the band intensity using ImageJ software. Band intensities of Ube3a **(B)**, HDAC1 **(C)**, and HDAC2 **(D)** were measured, divided by the band intensity of respective β-actin to normalize and expressed as fold change, while acetylated histones H3(K9) and H4(K12) were normalized against respective total histone (acetylated/total) and expressed as fold change. Values are mean ± SD with four animals in each group. The “a” indicate *p* < 0.05 compared to respective wild type group at E16, P5 and P60. Values were analyzed by two-way ANOVA with Holm–Sidak *post-hoc* test.

We have earlier demonstrated that the deficiency of Ube3a in AS mice brain results in aberrant increase in HDAC1/2 activities during the adult stage (Jamal et al., 2017). But how Ube3a regulates HDAC1/2 activities and how early aberrant increase in HDAC1/2 activities can be seen in AS mice brain is not known. Here, we first aimed to investigate the temporal pattern of altered HDAC1/2 activities in AS mice brain. Cortical brain region of AS mice along with their age-matched wild type controls were obtained from the early developmental period (E16), early post natal day (P5) and adult age (P60) and processed for immunoblot analysis of Ube3a, HDAC1/2 and total and acetylated histones H3(K9)/H4(K12). Figure 1 showed that the expression of Ube3a in the cortical region of AS mice reduced to about 70% at E16, 90% at P5 and undetectable at P60. The level of both HDAC1 and HDAC2 as well as acetylated histones H3(K9)/H4(K12) were decreased age-dependently in both wild type and AS mice cortical samples. However, HDAC1/2 levels were found to be significantly elevated in AS mice cortex from E16 stage and remained up-regulated up to the adult stage when compared with age-matched wild type control group. Acetylated derivatives of histones H3(K9) and H4(K12) were found to be significantly decreased in AS mice cortex samples from E16 stage. These results indicate that levels and activities of HDAC1/2 are increased in AS mice brain at least from E16 stage.

### Regulation of HDAC1/2 Activities by Ube3a

**FIGURE 2:**
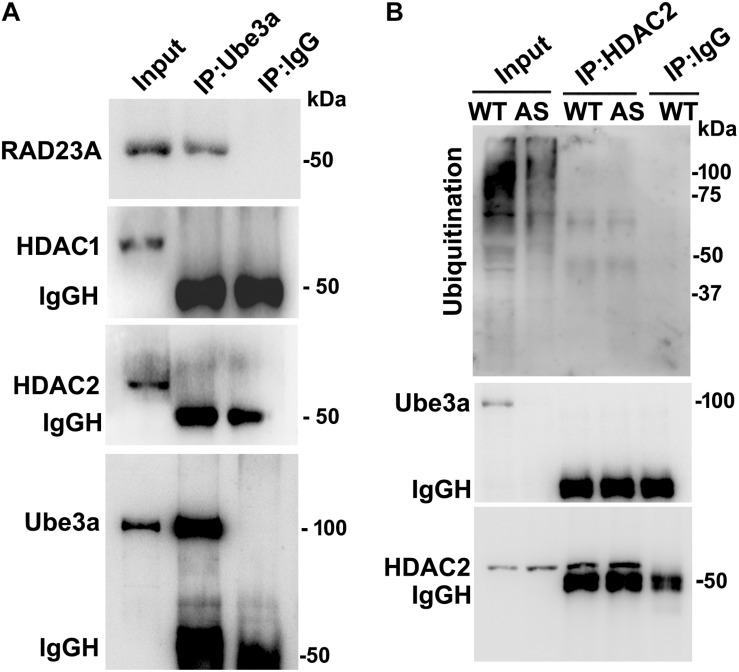
Ube3a does not interact with either HDAC1 or HDAC2. **(A)** Cortical brain lysates from the wild type mice were co-immunoprecipitated with Ube3a antibody (mouse specific from Sigma) and blots were probed with Ube3a, HDAC1, HDAC2 and RAD23A antibodies. RAD23A was used as a positive control that interacted with Ube3a. **(B)** Brain lysates were co-immunoprecipitated by HDAC2 antibody (mouse specific from Cell Signalling Technology) and blots were detected with HDAC2, Ube3a and ubiquitin antibodies.

**FIGURE 3:**
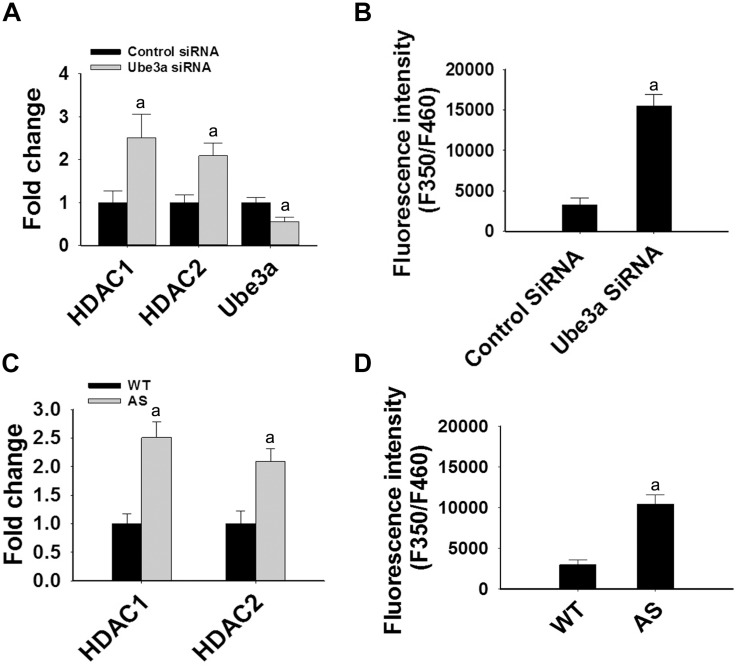
Ube3a deficiency causes significant up-regulation in the transcript level of both HDAC1/2 and HDAC enzymatic activity. **(A)** HT22 cells were transiently transfected with Ube3a and control siRNA for 24 h, total RNA was extracted, cDNA was synthesized and then subjected to RT-qPCR analysis of Ube3a, HDAC1 and HDAC2 transcripts. **(B)** Measurement of HDAC enzymatic activity in the crude nuclear extract prepared from control and Ube3a siRNA transfected cells as described in materials and methods. Values are mean ± SD of four different experiments. **(C)** Cortices from wild type and AS mice (P60) were isolated, total RNA was extracted and processed for RT-qPCR. **(D)** HDAC enzymatic activity in cortical samples of adult (P60) wild type and AS mice. Expression levels of HDAC1 and HDAC2 mRNAs were calculated and normalized to 18S rRNA (HDAC/18 SrRNA) as an internal control. Values plotted are mean ± SD of four mice in each group. The “a” depict *p* < 0.001 compared to respective control siRNA **(A,B)** or wild type mice **(C,D)** group (Students *t-*test).

Since levels and activities of HDAC1/2 are increased in AS mice brain, we further aimed to study Ube3a-mediated regulation of HDAC1/2 levels. As Ube3a acts as an ubiquitin ligase, we initially checked the interaction of HDAC1 and HDAC2 with Ube3a. Cortical lysates from wild type mice (P60) were co-immunoprecipitated with Ube3a antibody and blots were probed with antibodies against HDAC1, HDAC2 and RAD23A. Ube3a did not interact with either HDAC1 or HDAC2 but showed clear interaction with RAD23A that was used as a positive control (Figure 2A). In reverse co-immunoprecipitation assay, brain lysates were immunoprecipitated by HDAC2 antibody and blots were detected with Ube3a and ubiquitin antibodies. In this case also we did not find interaction between HDAC2 and Ube3a (Figure 2B). The ubiquitination profile of HDAC2 in AS sample was also very similar like wild type sample (Figure 2B). Next, we analyzed the half-life of HDAC1 and HDAC2 in the presence and absence of Ube3a. HT22 cells were transiently transfected with control and Ube3a siRNA plasmids for 24 h and chased for different time periods in the presence of cycloheximide. Collected cells were then subjected to immunoblot analysis using HDAC1 and HDAC2 antibodies. We observed that the partial knock down of Ube3a did not alter the half-life of either HDAC1 or HDAC2 (Supplementary Figure S1). This indicates that HDAC1 and HDAC2 are not targeted by Ube3a for their proteasomal degradation. Next, we explored co-activator role of Ube3a in regulating HDAC1/2 levels. Ube3a was transiently knocked down in HT22 cells and then checked for transcript levels of both HDAC1/2. As shown in Figure 3A, partial knockdown of Ube3a resulted in significant increase in the transcription of both HDAC1/2. We further measured the enzymatic activity of total HDAC in the nuclear extract of Ube3a and control siRNA transfected cells and detected a considerable increase in HDAC activity in the Ube3a knockdown cell (Figure 3B). We next analyzed transcript levels of both HDAC1/2 in AS mice cortical samples along with wild type control and detected a significant increase in their transcript levels in AS mice samples when compared to wild type control (Figure 3C). The enzymatic activity of total HDAC was also significantly higher in AS mice cortical samples in comparison with wild type control (Figure 3D). These results suggest that Ube3a regulates transcription of both HDAC1/2 and increased HDAC1/2 levels and activities in AS mice brain are due to their transcriptional up-regulation.

### Treatment of Simvastatin Restores Decreased HDAC Activity as Well as BDNF Level in HT22 Cells

**FIGURE 4:**
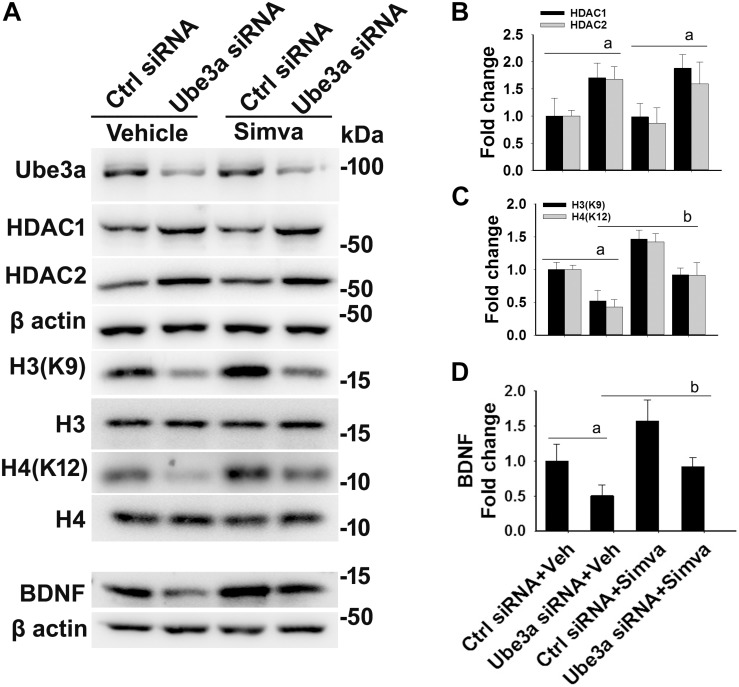
Simvastatin treatment restores histones H3/H4 hypo-acetylation and altered BDNF level in Ube3a-deficient HT22 cells. **(A)** HT22 cells were transfected with control and Ube3a siRNA. Twenty four hours of post transfection, cells were treated with either vehicle or 5 μM simvastatin for 12 h. Cells were harvested and processed for immunoblotting using antibodies against Ube3a, HDAC1, HDAC2, total and acetylated histones H3/H4 and BDNF. **(B–D)** Band intensities of HDAC1/2 **(B)**, acetylated histones H3/H4 **(C)**, and matured BDNF **(D)** were quantified and plotted as described in Figure 1. Values are mean ± SD of three independent experiments. Data were analyzed by one-way ANOVA with Holm–Sidak *post hoc* test. The ‘a’ signify *P* < 0.01 compared to vehicle or simvastatin treated control siRNA group and “b” represent *P* < 0.01 with regard to vehicle treated Ube3a siRNA group.

The aberrantly increased HDAC1/2 activities in AS mice brain led us to test the possible impact of HDAC inhibitors in reversing AS phenotypes. Recently, we have shown HDAC inhibitor, sodium valproate, partially restored behavioral deficits in AS mice (Jamal et al., 2017). Here we attempted to test the effect of simvastatin, as this widely used brain permeable cholesterol lowering drug, not only shown to inhibit HDAC1/2 activities but also induces the expression of BDNF (Lin et al., 2008; Roy et al., 2015). The BDNF is well known to regulate the synaptic plasticity and is significantly decreased in AS mice brain (Bramham and Panja, 2014; Jamal et al., 2017). First, we have tested the effect of simvastatin on HDAC activity and BDNF expression in HT22 hippocampal cell line. We found that this drug significantly reduced the HDAC activity as evident from the increased acetylation of histones H3(K9) and H4(K12) without affecting its level and these findings are consistent with the earlier report (Lin et al., 2008). Simvastatin also dose-dependently increased the expression of BDNF in HT22 cells (Supplementary Figure S2). Interestingly, we observed that the treatment of simvastatin rescued the reduced acetylation of histones H3(K9)/H4(K12) as well as expression of BDNF in Ube3a-deficient HT22 cells (Figure 4).

### Oral Administration of Simvastatin in AS Mice Rescues Altered HDAC Activity and Improves Behavioral Deficits in AS Mice

**FIGURE 5:**
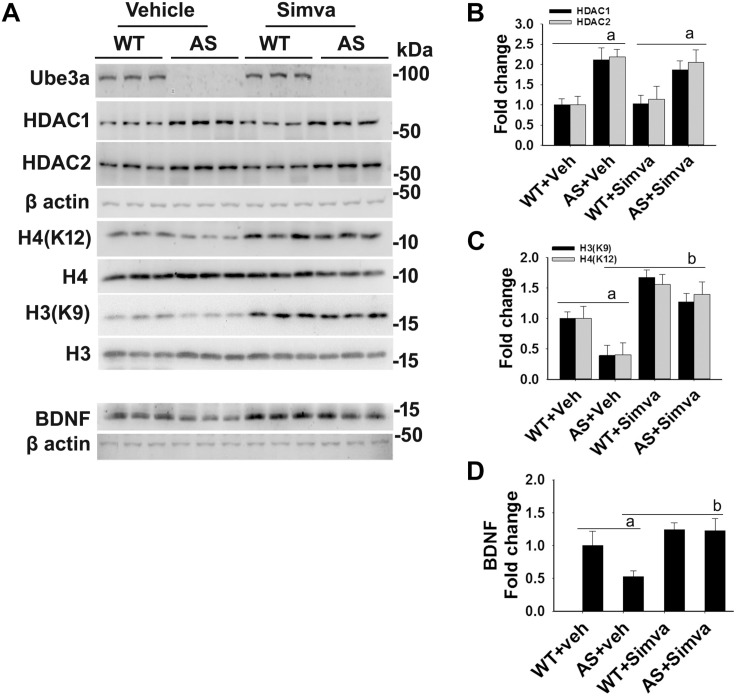
Oral administration of simvastatin significantly recovers the reduced acetylation of histones H3/H4 and altered level of BDNF in AS mice brain. **(A)** Adult wild type and AS mice were orally administered simvastatin (1 mg/kg body weight) for 60 days, cortices were dissected out, lysed and subjected to immunoblotting using antibodies against Ube3a, HDAC1/2, histones H3/H4 and their acetylated derivatives and BDNF. **(B–D)** Quantitation of band intensities of HDAC1/2 **(B)**, acetylated histones H3/H4 **(C)**, and matured BDNF **(D)** as described in Figure 1. Each lane represents sample from the different mouse. Values plotted are mean ± SD with four mice in each treatment group. Data were examined by one-way ANOVA followed by Holm–Sidak *post hoc* test. The ‘a’ represent *P* < 0.01 compared to vehicle or simvastatin treated wild type mice group while “b” denote *P* < 0.01 with respect to vehicle treated AS mice group.

**FIGURE 6:**
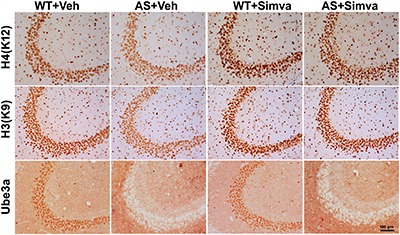
Representative immunohistochemical staining of acetylated histones H3(K9) and H4(K12) in the hippocampal CA3 region of wild type and AS mice received simvastatin or vehicle. Brain sections (20 μm thickness) collected from all four different groups of mice were placed on the same slide and processed for immunostaining of H3(K9), H4(K12) and Ube3a. Sections from four different mice in each experimental group were evaluated. Hippocampal CA3 area is shown in the image. Scale bar, 100 μm.

**FIGURE 7:**
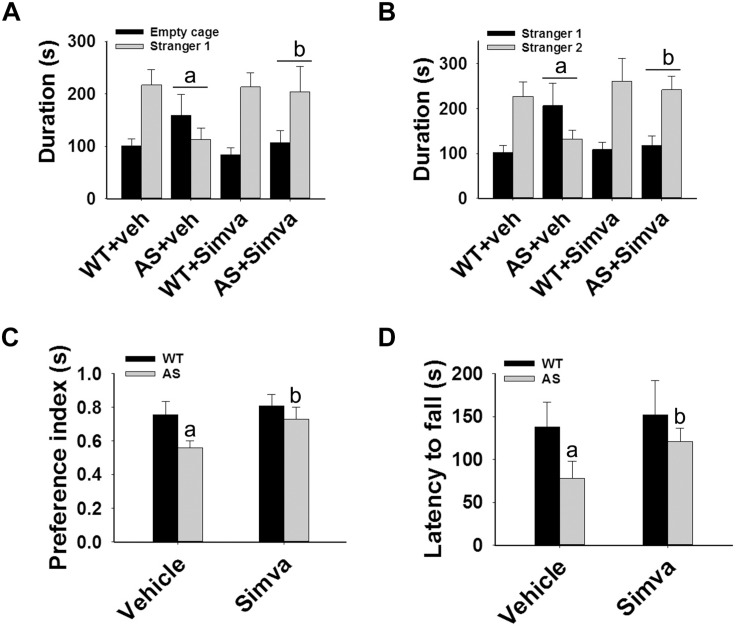
Oral administration of simvastatin enhances social interaction, improves memory deficit and motor coordination in AS mice. Wild type and AS mice were orally given either vehicle (DMSO) or simvastatin (1 mg/kg body weight) for 60 days. Behavioral tests were performed between 45 and 55 days of simvastatin treatment. **(A,B)** Test for social interaction displaying significant enhancement of sociability **(A)** and social novelty **(B)** in AS mice in response to simvastatin. **(C)** Novel object recognition test showing significant increase in the preference for the novel object in AS mice upon drug exposure. **(D)** Motor coordination test. Values are mean ± SD with 10 animals in each group. The “a” indicate *P* < 0.05 compared to vehicle treated wild type group whereas “b” denote *P* < 0.01 compared to vehicle treated AS mice group. Data was analyzed by two-way **(A,B)** or one-way **(C,D)** ANOVA with Holm–Sidak *post hoc* test.

The restoration of altered HDAC1/2 activities as well as BDNF expression in Ube3a-deficient HT22 cells upon treatment with the simvastatin prompted us to test the effect of this drug on AS mice. Adult AS mice along with their age-matched wild type controls were administered simvastatin orally (1 mg/kg body weight daily for 60 days), sacrificed and collected brain samples were processed for immunoblot and immunohistochemical analysis of HDAC1 and HDAC2, acetylated histones and BDNF. Figure 5 showed that the prolonged treatment of simvastatin in AS mice restored hypo-acetylation of histones H3(K9)/H4(K12) nearly to wild type level in their cortical areas. HDAC1/2 levels that were significantly increased in AS mice brain remains unaffected upon treatment with simvastatin. The reduced level of matured BDNF was also normalized in AS mice cortical areas after treatment with simvastatin (Figure 5). Immunonohistochemical studies further revealed that the treatment of simvastatin caused partial restoration of acetylated histones H3(K9) and H4(K12) in the hippocampus and other regions of AS mice brain (Figure 6 and Supplementary Figure S3 for smaller magnification images). Simvastatin treatment did not alter the level of total histones H3 and H4 (Supplementary Figure S4) and this results were similar with the immunoblot data. Quantitation of immunohistochemical staining images of entire hippocampus showed about 40–50% reduction in the level of H3(K9) and H4(K12) in AS mice and treatment of simvastatin restored this level to about 5–15%. We have also conducted various behavioral tests during 45–55 days of simvastatin treatment. In the social interaction assessment, AS mice demonstrated significant difficulties in sociability (interaction with stranger 1 with respect to empty cup) and social novelty (interaction with stranger 2 with regard to stranger 1), whereas prolonged simvastatin administration substantially reversed these deficits (Figures 7A,B). In novel object recognition test, simvastatin administration also significantly improved the preference for novel object in AS mice when comparison with the vehicle treated AS group (Figure 7C). This result indicates that simvastatin has the potential to restore the impaired visual recognition memory in AS mice. Notably, prolonged treatment of simvastatin likewise improved the motor coordination in AS mice in rotarod performance test (Figure 7D). Thus, rescue of the behavioral phenotype in AS mice is correlated well with restoration of HDAC1/2 activities and BDNF level in response to simvastatin.

### Treatment of Simvastatin in Primary Cortical Neuronal Culture Made From AS Mice Embryo Restores Altered HDAC Activity and BDNF Level

**FIGURE 8:**
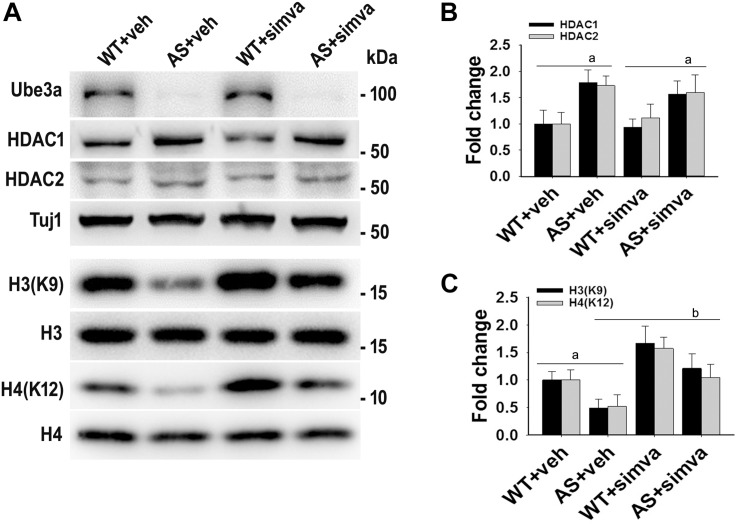
Simvastatin treatment restores acetylation of histones H3(K9)/H4(K12) in cultured primary cortical neurons prepared from AS mice embryo. **(A)** Mouse cortical primary neurons were cultured from the E16 embryo of time pregnant AS mice and maintained in neuronal maintenance media as described in materials and methods. Fourteen days *in vitro* (DIV14) neurons were treated with 5 μM simvastatin for 12 h. Collected cells were then processed for immunoblotting with antibodies against Ube3a, HDAC1/2 and total and acetylated histones H3/H4. **(B,C)** Band intensities of HDAC1/2 **(B)** were normalized to Tuj1 (HDAC/Tuj1) while acetylated histones H3/H4 **(C)** were normalized to their respective total histones (acetylated/total). Values plotted are mean ± SD of three independent experiments. The ‘a’ signify *P* < 0.05 compared to vehicle or simvastatin treated wild type group while “b” denote *P* < 0.05 with respect to vehicle treated AS group (one way ANOVA with Holm–Sidak *post hoc* test).

**FIGURE 9:**
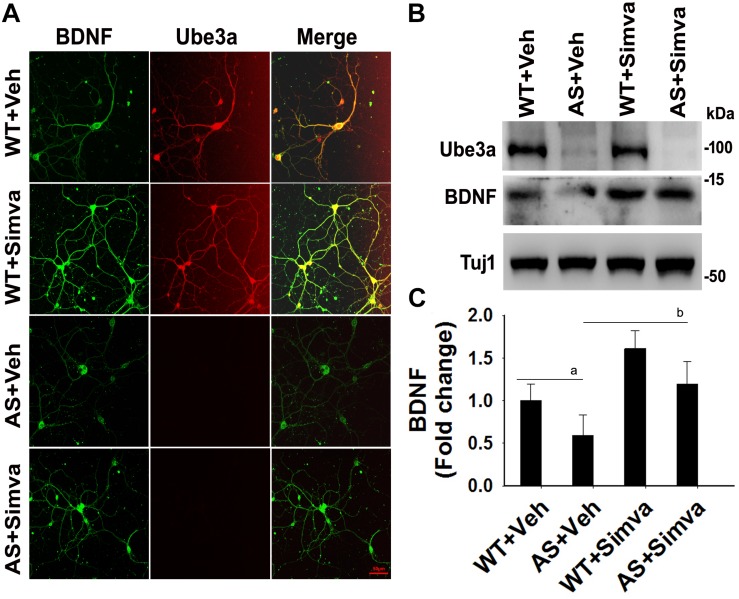
Simvastatin treatment increases BDNF expression in primary cortical neurons of AS mice. **(A)** Primary cultured cortical neurons prepared from wild type and AS embryos were treated with 5 μM simvastatin at DIV14 for 12 h. Neurons were then fixed and processed for double immunofluorescence staining using Ube3a and BDNF antibodies. Representative images were shown. About 12 immunostained neurons in each group were checked for BDNF expression, fluorescence intensity in each cell was quantified and compared. Scale bar, 50 μm. **(B)** Immunoblot analysis of matured BDNF levels in simvastatin treated primary cortical neurons as described above. **(C)** Band intensity of the mature BDNF was quantified and normalized to Tuj1 (BDNF/Tuj1). Values are mean ± SD; *n* = 3. The ‘a’ point *P* < 0.05 compared to vehicle treated wild type group and “b” denote *P* < 0.05 with respect to vehicle treated AS group (one way ANOVA with Holm–Sidak *post hoc* test).

To further support our findings and to better understand about the role of simvastatin in modulating neuronal function, we treated simvastatin to primary cortical neuronal culture prepared from both wild type and AS mice embryo at E16. Simvastatin was treated to DIV14 cortical neurons for 12 h and then assessed for restoration of HDAC1/2 activities as well as the level of BDNF. As shown in Figure 8, DIV14 primary cortical neurons of AS mice exhibited significantly elevated levels of HDAC1 and HDAC2 along with reduced acetylation of histones H3(K9) and H4(K12). Treatment of simvastatin nearly normalized the reduced acetylation of both histones H3(K9) and H4(K12) in the primary cortical neuron of AS mice. The level of BDNF in primary cortical neurons (prepared from both wild type and AS mice embryo) was also assessed by immunoblotting as well as immunofluorescence staining. Primary cultured cortical neurons of AS mice at DIV14 showed about 40–45% reduced level of BDNF when compared to wild type control group and the treatment of simvastatin brought it around normal level in both immunostaining and immunoblot analysis experiments (Figure 9). These results are very similar with the *in vivo* animal experimentation data, which clearly indicate the potential role of simvastatin in restoring the altered HDAC1/2 activity and thereby rescuing behavioral deficits in AS mice.

## Discussion

In this manuscript, we first demonstrate that Ube3a regulates the transcription of both HDAC1 and HDAC2 and the loss of function of Ube3a in AS mice cause significant up-regulation of HDAC1/2 in their brain from the early developmental period. In the second set of experiments, we find that oral administration of simvastatin results in rescue of various behavioral deficits in AS mice along with restoration of altered HDAC1/2 activities and reduced expression of the BDNF. Our results also show that the treatment of simvastatin in primary cortical neuronal culture obtained from AS mice embryos potentially restore altered activities of HDAC1/2 and reduced level of BDNF.

Recently, we have shown aberrantly increased HDAC1/2 levels as well as hypo-acetylation of histones H3/H4 in different brain regions of adult AS mice (Jamal et al., 2017). However, the probable cause in their up-regulation was not clear. Our results suggest that Ube3a does not directly regulate the turnover of either HDAC1 or HDAC2. Conversely, we observed a transcriptional regulation of HDAC1/2 by Ube3a and in AS mice brain up-regulated HDAC1/2 can be seen at least from E16. At E16 stage, expression of paternal Ube3a was reduced to about 70%, indicating further that Ube3a deficiency is closely involved in regulating the expression of both HDAC1/2. Ube3a could target proteasome-mediated degradation of some of the transcription factors/co-activators involved in the regulation of either HDAC1 or HDAC2 expression. For example, Ube3a is reported to act as a co-activator of glucocorticoid receptor (GR) and at the same time it targets the liganded GR for proteasomal degradation (Godavarthi et al., 2012). Increased GR level was also observed in different brain regions of AS mice from very early post natal days (Godavarthi et al., 2014). Furthermore, GR has been shown to bind to the proximal promoter region of HDAC2 and positively modulates its activity (Graff et al., 2012). Alternately, Ube3a could also function as co-repressor of HDAC1 or HDAC2 like mSin3a or Co-REST (Guan et al., 2009). In any case, increased activities of both HDAC1/2 in AS mice brain could be potentially linked with altered synaptic function and plasticity along with associated behavioral deficits, including learning and memory and social interaction. Increased HDAC1/2 activities in the early developmental stage also could lead to defects in the activity-dependent synaptic plasticity observed in AS mice (Yashiro et al., 2009; Sato and Stryker, 2010; Kim et al., 2016). Multiple reports have demonstrated that HDAC2 negatively controls synaptic function and plasticity upon binding with the promoter of various plasticity and memory-related genes (Guan et al., 2009; Bousiges et al., 2010; Yamakawa et al., 2017). HDAC2 deficiency also shown to affect critical period of plasticity in mouse visual cortex (Nott et al., 2015). We have shown significant down-regulation of BDNF and synaptophysin in the hippocampus of AS mice in comparison with the wild type group (Jamal et al., 2017). Comprehensive analysis of all synaptic plasticity related genes that are regulated by either HDAC1 or HDAC2 would provide further insight in this regard.

Interestingly, we found that oral administration of simvastatin to AS mice significantly improved their memory deficit and social interaction and these improvements might be due to the restoration of at least HDAC1/2 activities. We further noted that simvastatin treatment in primary cortical neuronal culture as well as in HT22 hippocampal cell line considerably increased the expression of BDNF along with restoration of acetylated histones H3/H4. In addition to inhibiting popularly known HMG CoA reductase enzyme, statins are also shown to inhibit activities of HDAC1/2 through direct binding in their catalytic site (Lin et al., 2008). Furthermore, statins also stimulate the expression of BDNF, which could be mediated via HDAC1/2 as well as peroxisome proliferator-activated receptor alpha (PPARα) (Roy et al., 2015). As simvastatin inhibits the activity of HDAC1/2, one could expect restoration of various synaptic plasticity related genes in AS mice that eventually could rescue the behavioral deficit. A recent report has shown that the treatment of lovastatin reduced the seizure and hyper excitability in AS mouse model, although the mechanistic basis was unexplored (Chung et al., 2018). Our findings not only provide a possible explanation for the reduced seizure and hyper excitability but also restoration of other behavioral deficits in these mice. Simvastatin or lovastatin could be the better choice of drug in rescuing behavioral deficits in AS when compared with other HDAC inhibitors like sodium valproate (Jamal et al., 2017).

A growing body of evidence now advocates that statins could be promising drug for the treatment of various neuropsychiatric disorders. Statins are implicated in rescuing cognitive dysfunction in various animal models of neurodevelopmental disorders like Rett syndrome, Fragile X syndrome, neurofibromatosis type 1 etc. (Li et al., 2005; Buchovecky et al., 2013; Osterweil et al., 2013; van der Vaart et al., 2013). Statins are also shown to improve the cognitive function in mouse models of Alzheimer’s disease (Tong et al., 2012; Roy et al., 2015). However, there are conflicting reports regarding the use of statins and improvement of cognitive function in multiple case control studies, and clinical trials of neurodevelopmental as well as neurodegenerative disorders (Li et al., 2007; Sano et al., 2011; Richardson et al., 2013; Stivaros et al., 2018). Therefore, extensive studies are required to better understand the role of statin in regulating cognitive function.

Overall, our study finds that Ube3a regulates the transcription of HDAC1 and HDAC2 and aberrant increase in activities of these enzymes in AS mice brain might lead to defect in synaptic function and plasticity, the underlying cause of cognitive and other behavioral deficits in these mice. Our findings also suggest that simvastatin could be a promising drug to treat AS.

## Data Availability Statement

The datasets generated for this study are available on request to the corresponding author.

## Ethics Statement

The animal study was reviewed and approved by the Animal Ethic Committee of the National Brain Research Centre, India.

## Author Contributions

NJ conceived the study. VK, NV, TJ, and BS performed the experiments. VK and NV analyzed the data. NJ wrote the manuscript. All authors reviewed the manuscript.

## Conflict of Interest

The authors declare that the research was conducted in the absence of any commercial or financial relationships that could be construed as a potential conflict of interest.
