# Supplementary material for: Simvastatin Restores HDAC1/2 Activity and Improves Behavioral Deficits in Angelman Syndrome Model Mouse
Source: Front Mol Neurosci. 2019 Nov 26;12:289. doi: 10.3389/fnmol.2019.00289 (PMC6901934; doi:10.3389/fnmol.2019.00289)

Supplementary information

Supplementary Fig.S1. Ube3a does not affect the degradation of either HDAC1 or HDAC2. (A) HT22 cells were transiently transfected with control and Ube3a siRNA and 24h later cells were chased with cycloheximide (25 μg/ml) for different time periods indicated in the figure. Collected cells were then processed for immunoblot analysis using antibodies against Ube3a, HDAC1, HDAC2 and β-actin. (B, C) Quantitation of band intensities of HDAC1 and HDAC2 shown in A. Band intensities of HDAC1 (B) and HDAC2 (C) were normalized to β-actin and plotted.

Supplementary Fig.S2. Treatment of simvastatin to HT22 cells increased the acetylation of histone H3(K9) and H4(K12) as well as expression of BDNF. HT22 cells were treated with different doses of simvastatin for 12 h and then processed for immunoblot analysis using various antibodies indicated in the Figure. Band intensities were calculated, normalized (HDAC1 and HDAC2 with β-actin and acetylated histones with respective total histones) and expressed as fold change. Values plotted are mean ± SD of 3 independent experiments. The "a" represents $P<0.01$ in comparison with vehicle treated control group (one way ANOVA with Holm Sidak post hoc test).

Supplementary Fig.S3. Representative immunohistochemical staining of acetylated histones H3(K9) and H4(K12) in the hippocampal and associated cortical region of wild type and AS mice received simvastatin or vehicle. Brain sections collected from all 4 different groups of mice were placed on the same slide and processed for immunostaining of H3(K9), H4(K12) and Ube3a. Sections from 4 different mice in each experimental group were evaluated.

Supplementary Fig.S4. Representative immunohistochemical staining of total histones H3 and H4 in the hippocampal and associated cortical region of wild type and AS mice received simvastatin or vehicle. Brain sections obtained from all 4 different treatment groups were kept on the same slide and subjected to immunostaining using antibodies against total histones H3 and H4.

Figure S1

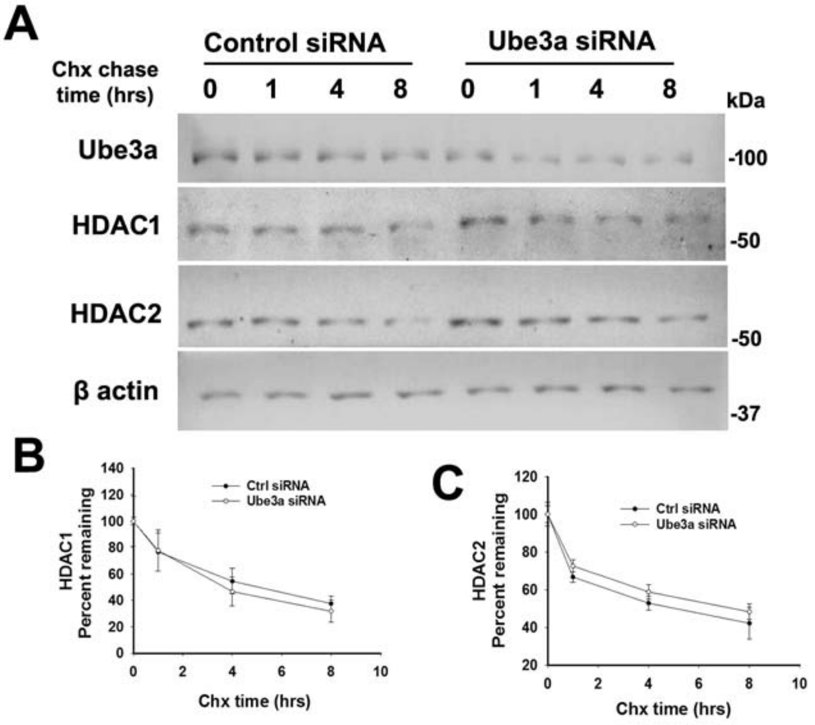
A
Control siRNA
Ube3a siRNA
Chx chase time (hrs)
0 1 4 8 0 1 4 8
kDa
Ube3a
-100
HDAC1
-50
HDAC2
-50
β actin
-37
B
HDAC1 Percent remaining
Ctrl siRNA
Ube3a siRNA
Chx time (hrs)
C
HDAC2 Percent remaining
Ctrl siRNA
Ube3a siRNA
Chx time (hrs)

Figure S2

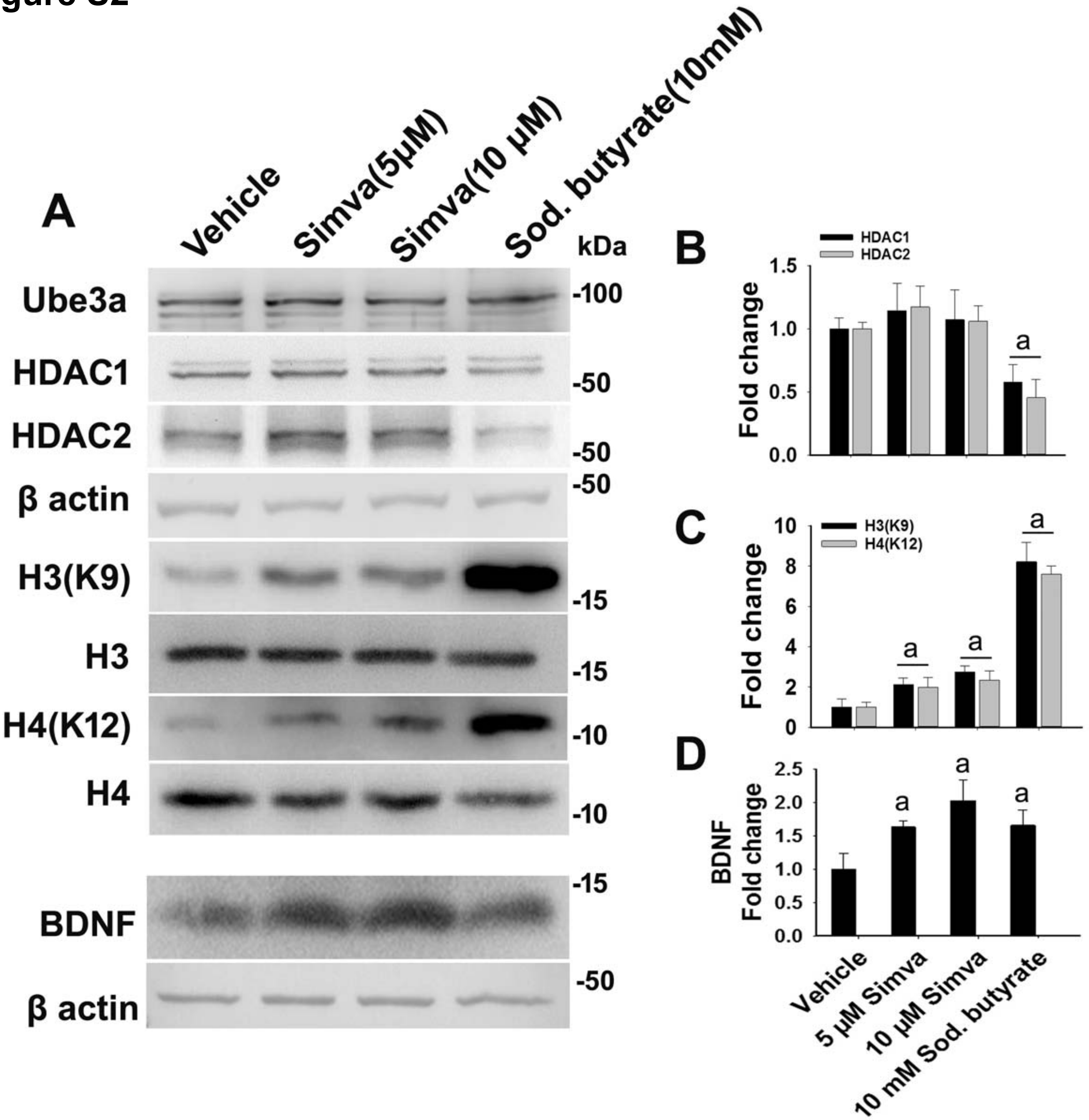
A
Vehicle
Simva(5μM)
Simva(10 μM)
Sod. butyrate(10mM)
kDa
Ube3a
HDAC1
HDAC2
β actin
H3(K9)
H3
H4(K12)
H4
BDNF
β actin
-100
-50
-50
-50
-15
-15
-10
-10
-15
-50
B
HDAC1
HDAC2
Fold change
C
H3(K9)
H4(K12)
Fold change
D
BDNF
Fold change
a
Vehicle
5 μM Simva
10 μM Simva
10 mM Sod. butyrate

Figure S3

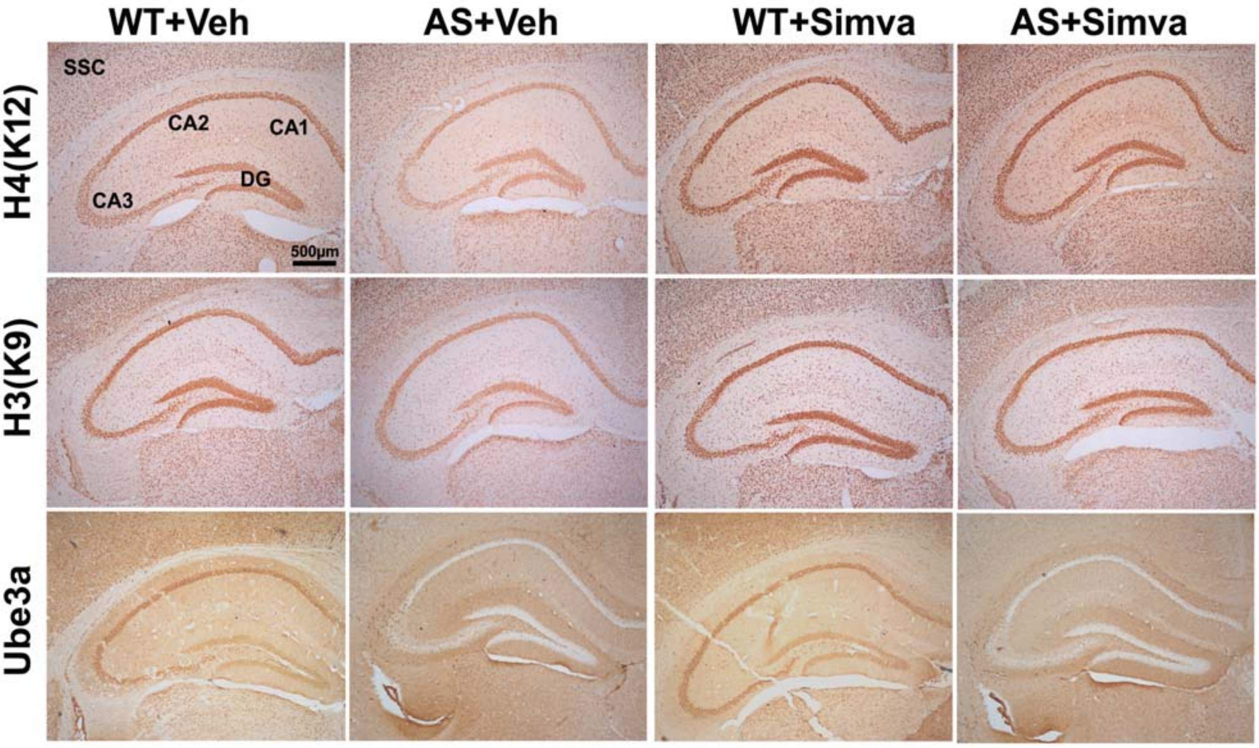

WT+Veh
AS+Veh
WT+Simva
AS+Simva
H4(K12)
H3(K9)
Ube3a
SSC
CA2
CA1
DG
CA3
500μm

Figure S4

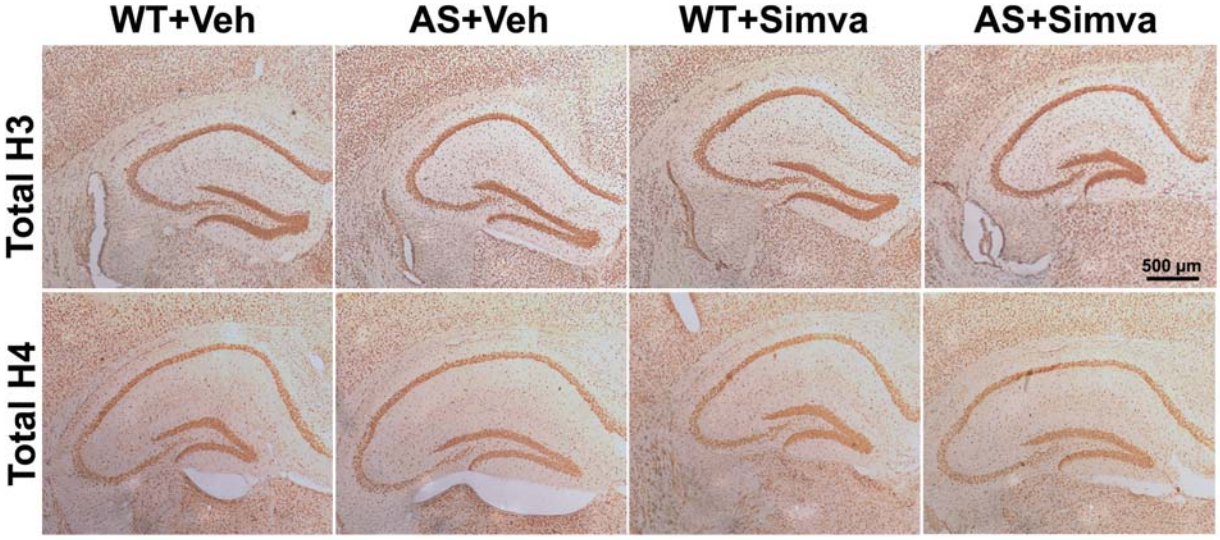

Supplement: Supplementary file 1 [file Data_Sheet_1.pdf]
